# Supplementary material for: Intermittent fasting attenuates glial hyperactivation and photoreceptor degeneration in a NaIO3-induced mouse model of age-related macular degeneration
Source: Commun Biol. 2025 Oct 1;8:1408. doi: 10.1038/s42003-025-08815-0 (PMC12488855; doi:10.1038/s42003-025-08815-0)
Supplement: Supplementary file 2 — Supplementary materials [file 42003_2025_8815_MOESM2_ESM.pdf]

Intermittent fasting attenuates glial hyperactivation and photoreceptor degeneration in a $NaIO_3$-induced mouse model of age-related macular degeneration

Jingzhen Li [a,b,1], Beibei Wang [a,b,1], Pinjie, Liu [a,b] , Xuecheng Qiu [a,b], Qiyun Bian [a,b], Congxin Shen [a,b], Yanyan Li [a,b], Mengwen Shao [a,b], Meng Li [a,b,*]

[a] *Jiangsu Key Laboratory of Brain Disease Bioinformation, Xuzhou Medical University, Xuzhou, Jiangsu 221000, China*

[b] *Department of Biochemistry, School of Basic Medical Sciences, Xuzhou Medical University, Xuzhou, Jiangsu 221000, China*

[1] These authors contributed equally to this work.

* Corresponding author. Jiangsu Key Laboratory of Brain Disease Bioinformation, Department of Biochemistry, School of Basic Medical Sciences, Xuzhou Medical University,

Address: 209 Tongshan Rd, Xuzhou, Jiangsu 221000, China.

E-mail address: limeng@xzhmu.edu.cn

Supplementary materials

Contents

Supplementary Figure S1. Retinal damage following various doses of $NaIO_3$ administration.

Supplementary Figure S2. Optomotor response (OMR) test under light and dark conditions.

Supplementary Figure S3. IF reduces $NaIO_3$-induced degeneration of RPE.

Supplementary Figure S4. IF reduces $NaIO_3$-induced degeneration of RPE.

Supplementary Figure S5. IF reduces $NaIO_3$-induced cell loss of photoreceptors.

Supplementary Figure S6. IF reverses $NaIO_3$-induced upregulation of apoptosis- and necrosis-related genes in the eye cups.

Supplementary Figure S7. IF attenuates $NaIO_3$-induced oxidative stress in the retina.

Supplementary Figure S8. Effects of IF on $NaIO_3$-induced RPE degeneration and microglial activation in 12-month-old mice.

Supplementary Figure S9. Effects of IF on $NaIO_3$-induced retina degeneration in 16-month-old mice.

Supplementary Figure S10. Raw images of Western blot.

Supplementary Table S1. list of reagents.

Supplementary Table S2. list of primary and secondary antibodies.

Supplementary Table S3. sequences of primers.

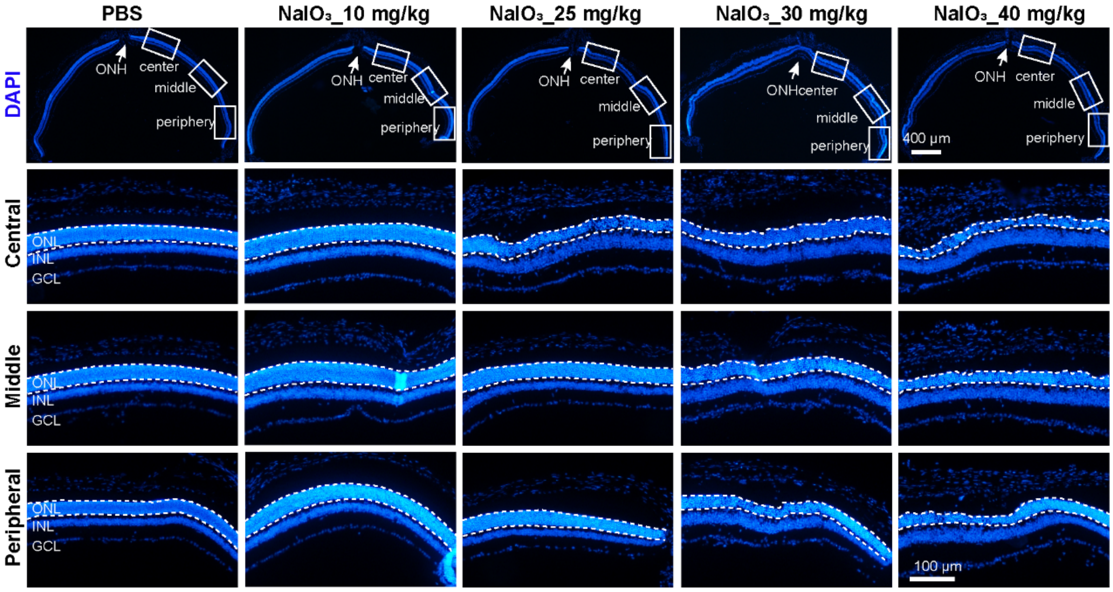

Supplementary Figure S1. Retinal damage following various doses of $NaIO_3$ administration. Nuclear staining with DAPI reveals structural changes in the retinas injected with 0, 10, 25, 30, or 40 mg/kg of $NaIO_3$. n=3 mice/group. ONH, optic nerve head; ONL, outer nuclear layer; INL, inner nuclear layer; GCL, ganglion cell layer. Scale bar, 400 μm (top row) or 100 μm (bottom three rows).

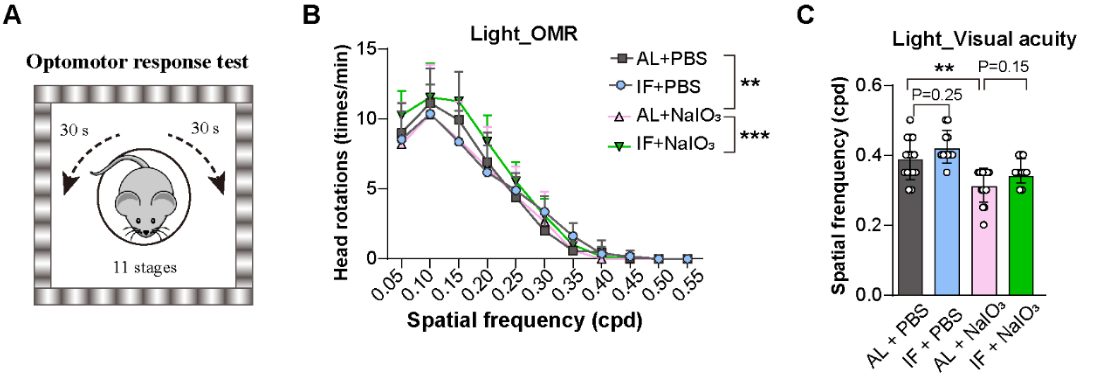

Supplementary Figure S2. Optomotor response (OMR) test under light and dark conditions. (A) Diagram of the OMR device used to test visual function. (B) Number of head rotation at different grating densities under light adaptation. n = 15 in AL+PBS，AL+$NaIO_3$ group and 11 in IF+PBS，IF+$NaIO_3$ group. Two-way ANOVA was used to calculate P values, revealing

significant differences in the average number of head movements across stages. (C) The grating density corresponding to the highest visual sensitivity (visual acuity) under light adaptation. Data are expressed as mean ± SD, P values were calculated using two-way ANOVA. ****P < 0.0001, **P < 0.01.

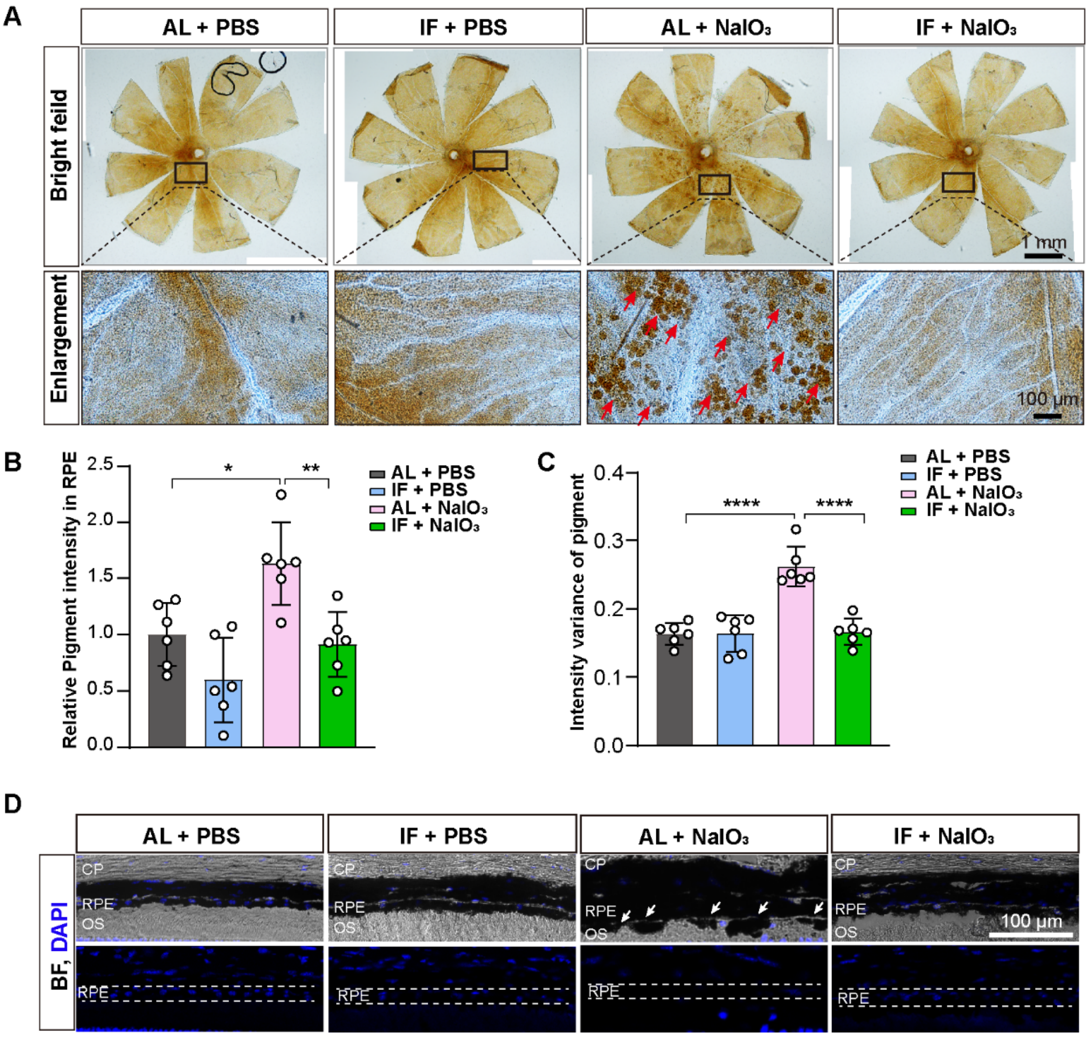

Supplementary Figure S3. IF reduces $NaIO_3$-induced degeneration of RPE. (A) Representative bright-field images of the pigment distribution in RPE flats of mice. Arrows indicate aggregated and fragmented pigment. Scale bar, 1 mm (top row) or 100 μm (bottom row). (B) Quantification of pigment intensity in RPE flat mounts. (C) The ratio of standard deviation to mean was calculated to reflect the fragmentation or uneven distribution of pigment

on RPE. n=6 mice/group. Data are expressed as the mean ± SD, P values were calculated using two-way ANOVA with Tukey HSD post hoc tests. ****P < 0.0001, **P < 0.01, *P < 0.05. (D) Representative images revealing the distribution of RPE pigments in retinal sections. Arrows indicate fragmented pigment in the RPE. n = 6 mice/group. Scale bar, 100 µm. Abbreviation: CP, choroid plexus; OS, outer segment.

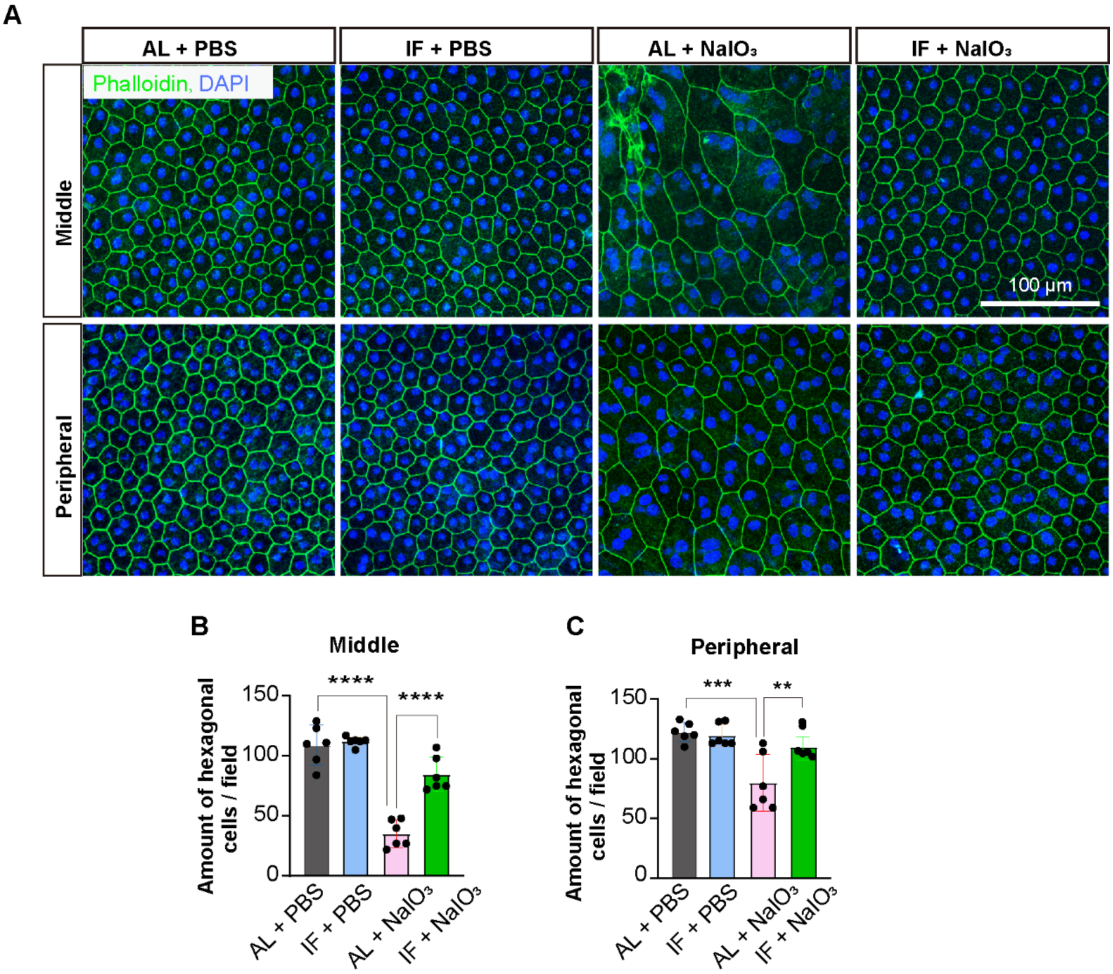

Supplementary Figure S4. IF reduces $NaIO_3$-induced degeneration of RPE. (A) Representative fluorescent images of phalloidin staining for F-actin and DAPI for nucleus showing morphology of RPE cells in the middle and peripheral regions of RPE whole mounts from 2-month-old mice. Scale bar, 100 µm. (B, C) Quantification of hexagonal cells in the middle and peripheral RPE regions. n = 6 mice/group. Data are expressed as the mean ± SD, P

values were calculated using two-way ANOVA with Tukey HSD post hoc tests in (B, C). **P < 0.01, ***P < 0.001, ****P < 0.0001.

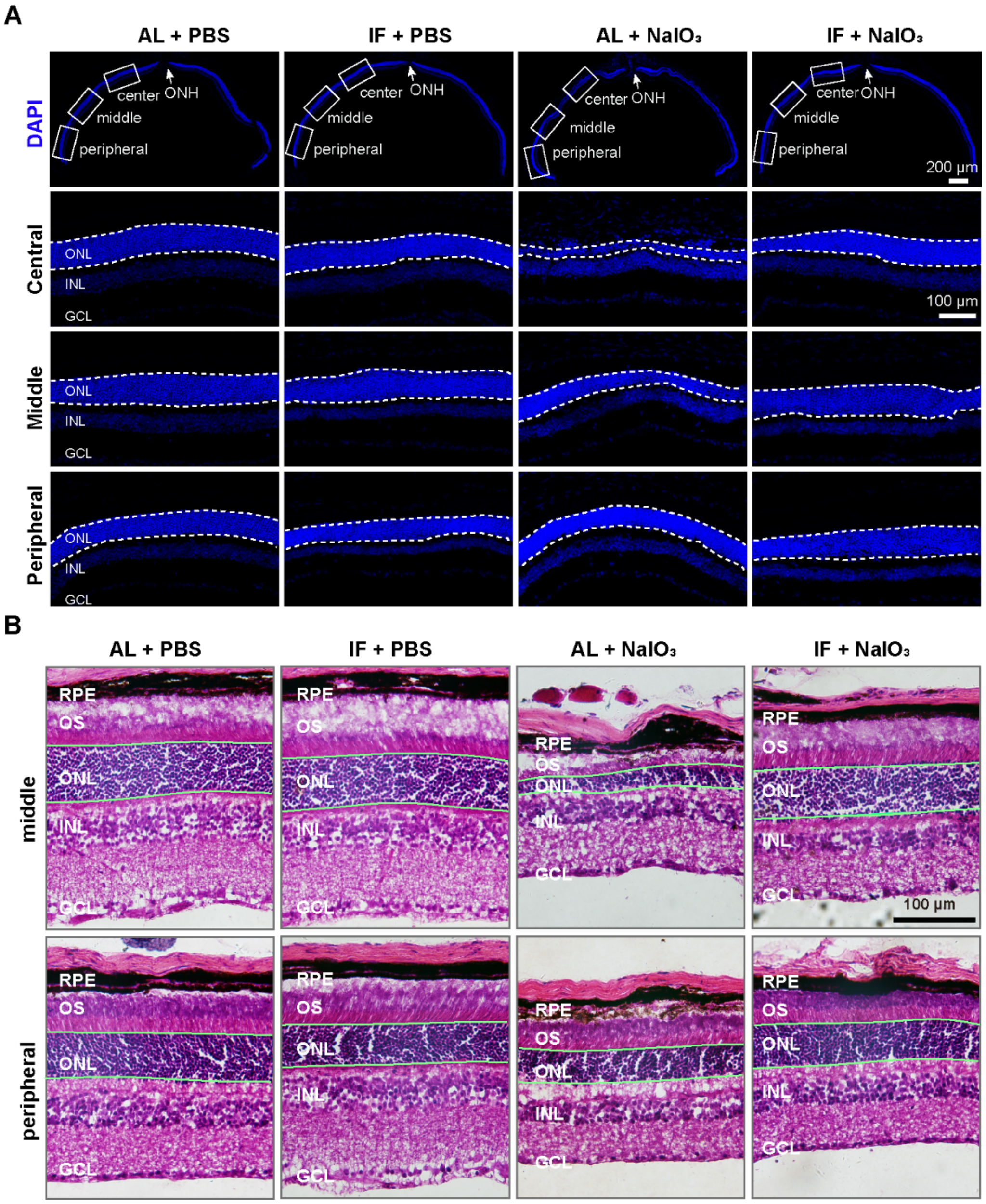

Supplementary Figure S5. IF reduces $NaIO_3$-induced cell loss of photoreceptors. (A) Representative images of DAPI staining revealing the nuclear layers of the retinas post $NaIO_3$ treatment. Scale bar, 100 µm (top row) or 200 µm (bottom three rows). n = 6 mice/group. (B) Representative images of H&E-stained retinal sections in the middle and peripheral regions. Scale bar, 100 µm. n = 6 mice/group.

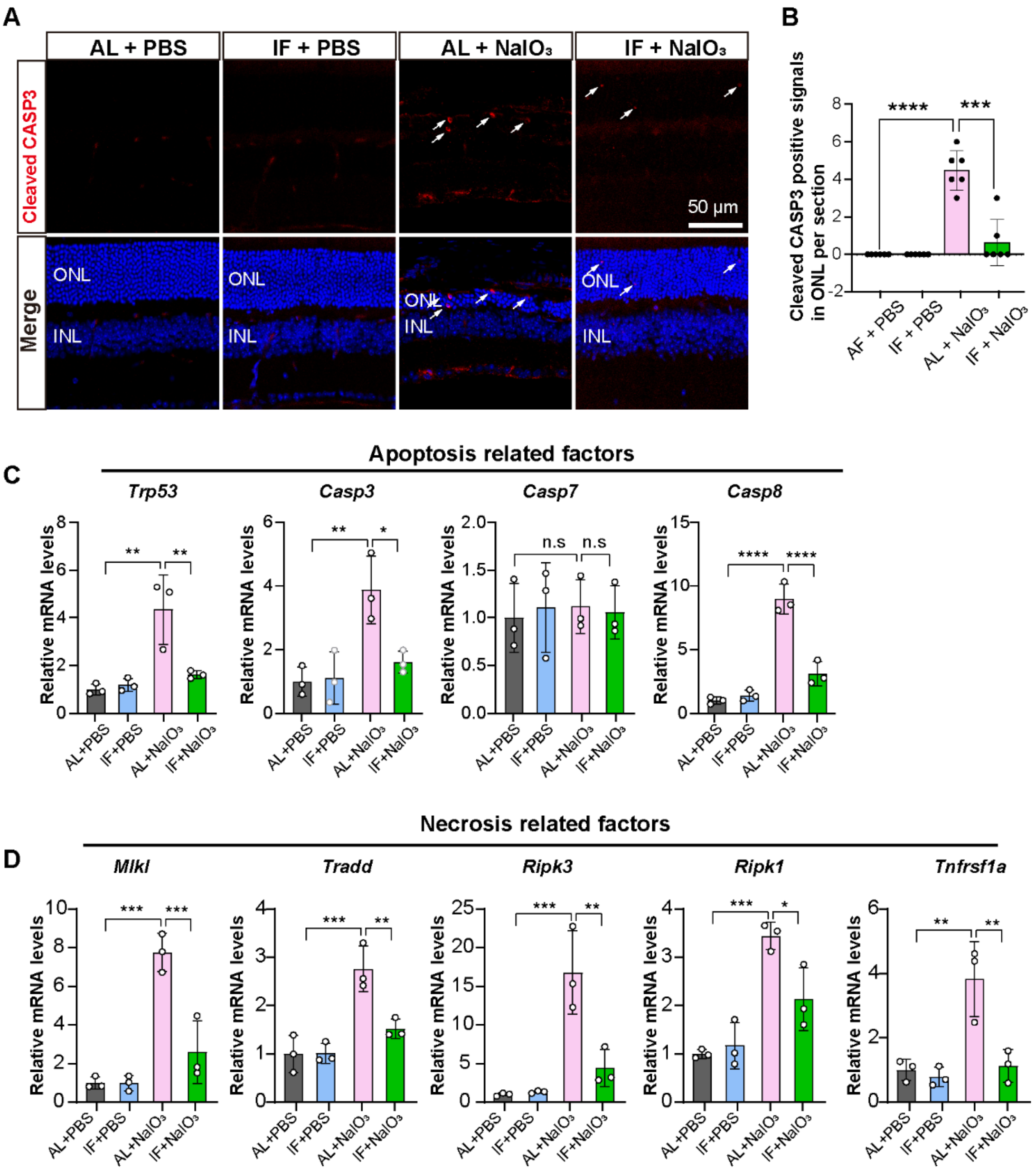

Supplementary Figure S6. IF reverses $NaIO_3$-induced upregulation of apoptosis- and necrosis-related genes in the eye cups. (A) Representative fluorescent images of cleaved-caspase3 signals in the retinal sections. Scale bar, 50 μm. (B) Quantification of cleaved-caspase3 fluorescence intensity. n=6mice/group. (C) qPCR analysis of mRNA levels of genes involved in cell apoptosis in the eye cups 7 days post $NaIO_3$ administration. *Gapdh*, *Actb*, *Ubc* were used as reference genes, n = 3 mice/group. (D) The mRNA levels of the genes involved

in cell necrosis in the eye cups as evaluated by RT-qPCR 7 days post-$NaIO_3$ administration. *Gapdh*, *Actb*, *Ubc* were used as reference genes, n = 3 mice/group. Data are expressed as the mean ± SD, P values were calculated using two-way ANOVA with Tukey HSD post hoc tests (B, C, D). ****P < 0.0001, ***P < 0.001, **P < 0.01, *P < 0.05.

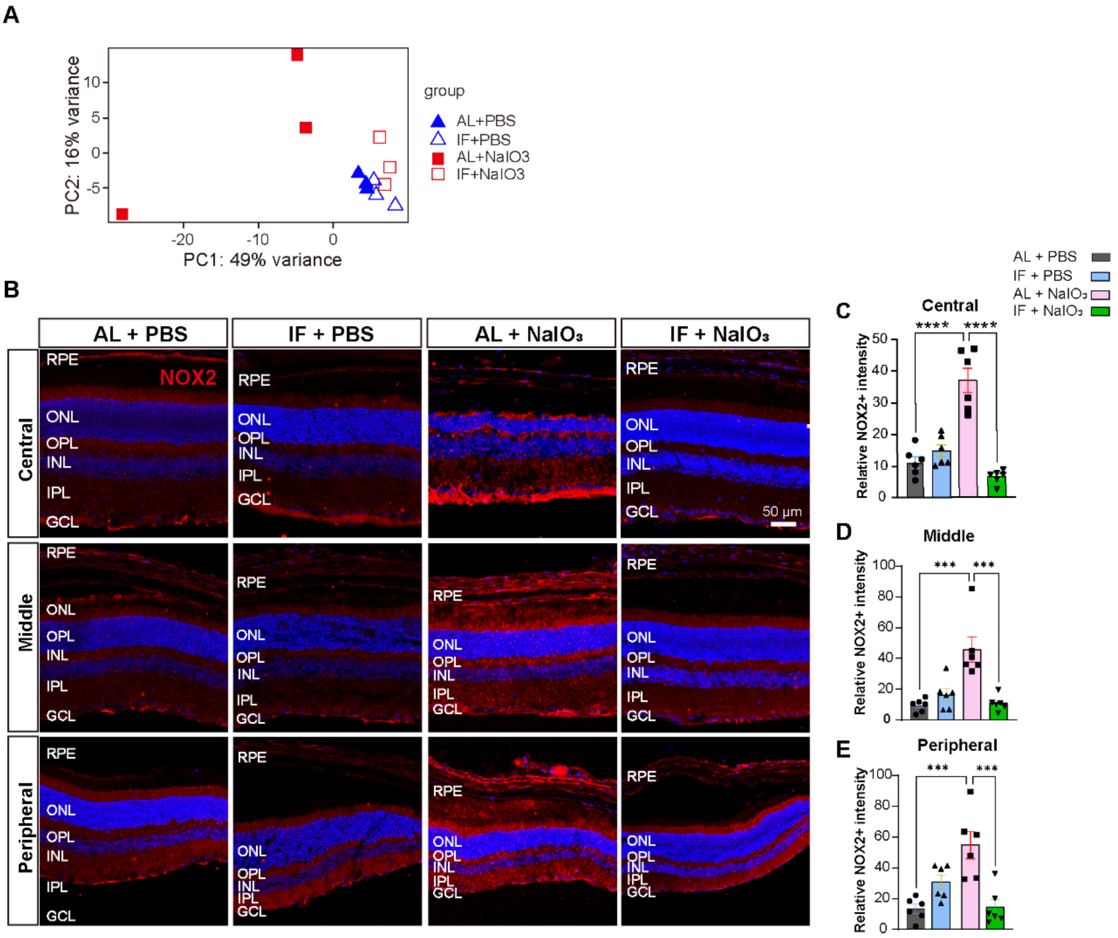

Supplementary Figure S7. IF attenuates $NaIO_3$-induced oxidative stress in the retina. (A) PCA (principal component analysis) plot is used to provide visualization of overall variance in transcriptomes between samples. (B) Representative fluorescent images of NOX2 in mice retina sections post $NaIO_3$ or PBS administration. Scale bar, 50 μm. (C-E) Quantification of NOX2 mean fluorescent intensity in the center, middle and peripheral regions. n = 6mice/group.

Data are expressed as the mean ± SD, P values were calculated using two-way ANOVA with Tukey HSD post hoc tests in (C, D). ***P < 0.001.

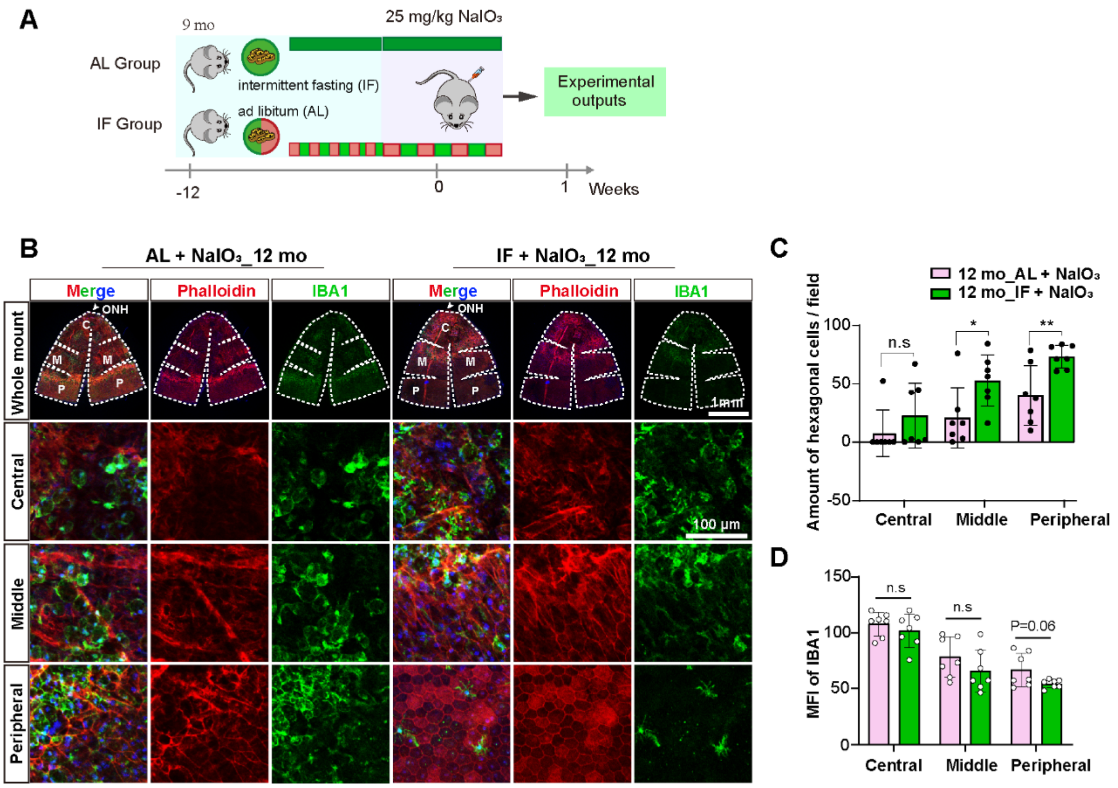

Supplementary Figure S8. Effects of IF on $NaIO_3$-induced RPE degeneration and microglial activation in 12-month-old mice. (A) Schematic of the experimental design. (B) Labeling of Phalloidin and IBA1 illustrating RPE integrity and microglia distribution in RPE flats. Scale bar, 1 mm (top row) or 100 μm (bottom three rows). (C, D) Quantification of hexagonal cell and IBA1 mean fluorescence intensity. n=7 mice/group. Data are expressed as mean ± SD, P values were calculated using unpaired two-tailed *t*-test in (C, D). **P < 0.01, *P < 0.05.

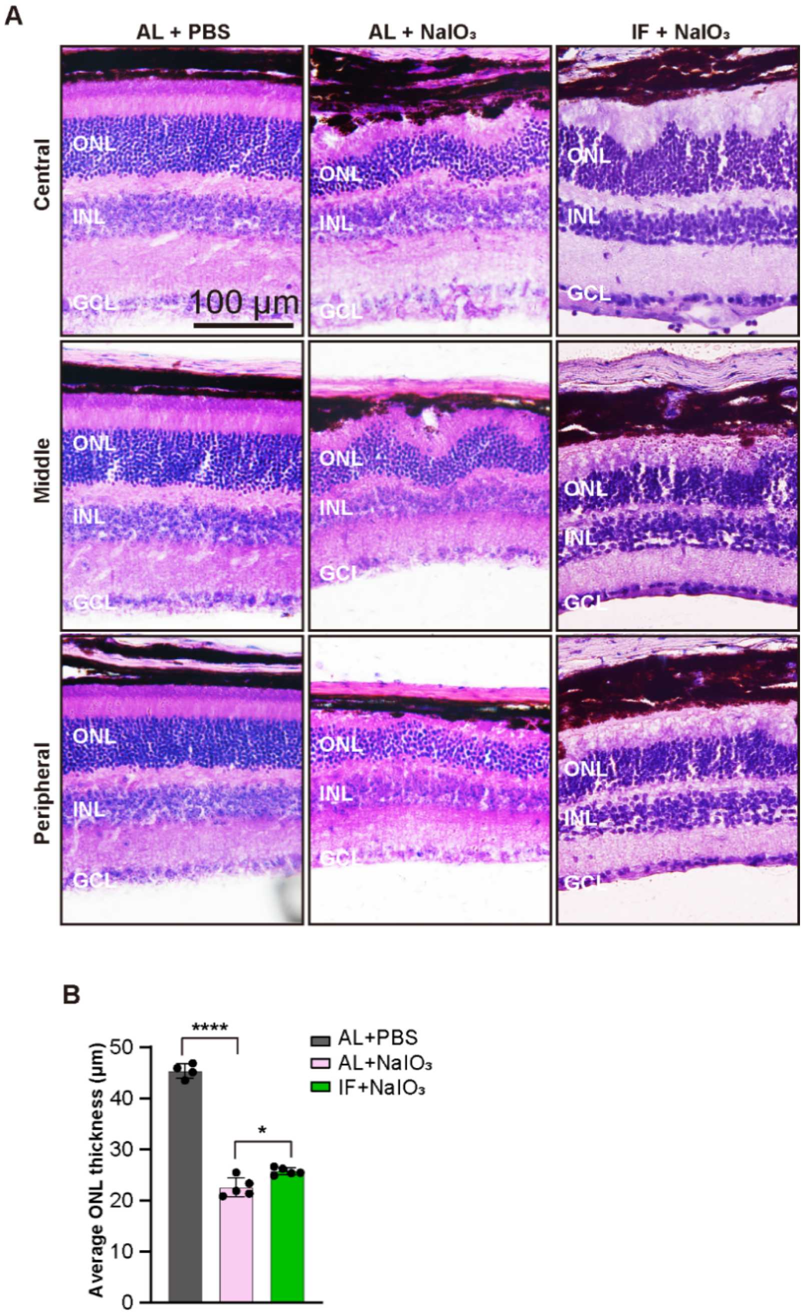

Supplementary Figure S9 Effects of IF on $NaIO_3$-induced retina degeneration in 16-month-old mice. (A) Representative images of DAPI staining revealing the nuclear layers of the retinas post $NaIO_3$ treatment. Scale bar, 200 µm. (B) Quantification of the average ONL thickess. n = 4 in AL+PBS and n=5 in AL+$NaIO_3$ and IF+$NaIO_3$. Data are expressed as the mean ± SD, P values were calculated using one-way ANOVA with Tukey HSD post hoc tests. *$P < 0.05$, ****$P < 0.0001$.

Figure 2B

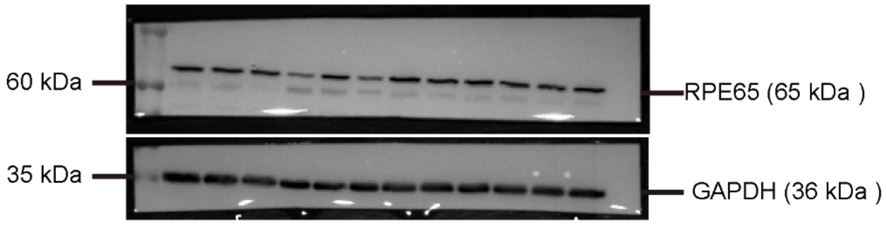

Figure 3J

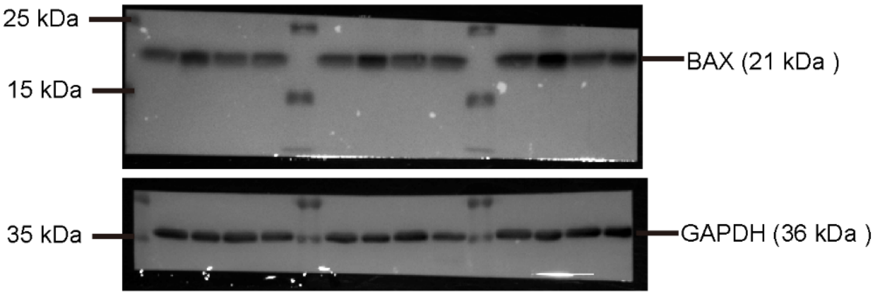

Figure 6K

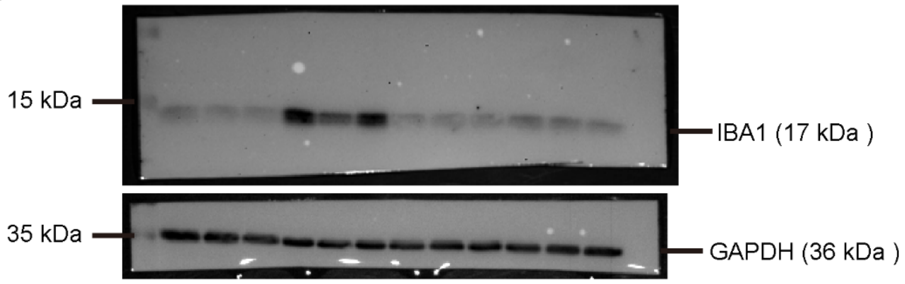

Figure 6K

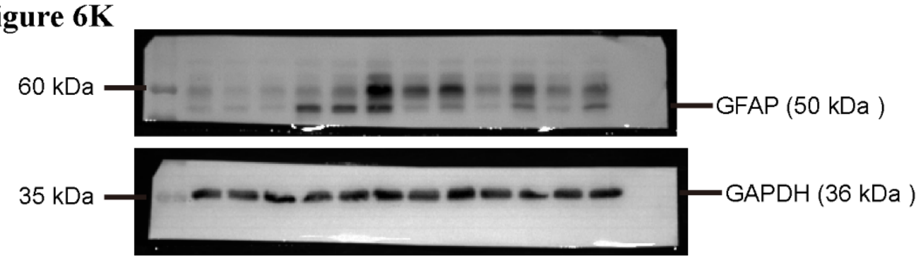

Supplementary Figure S10. Raw images of Western blot. **Note:** A single membrane was cut for Western blotting of RPE65 (Fig. 2B), IBA1 (Fig. 6K), and GAPDH as their shared loading control.

Supplementary Table S1: list of reagents.

Reagents	Manufacturer	Catalog number
$NaIO_3$	Sigma	S4007
Blood Keton kit	Solarbio	BC5060
Phalloidin	Beyotime	C2201S
PNA	MKBio	MP6327
Nile red	MKBio	MS4022
H&E staining	Beyotime	C0105S
TUNEL	Vazyme	A112-03
CM-H2DCFDA	MKBio	MX4827
FAS eye fixative solution	Servicebio	G1109
BCA protein assay kit	Beyotime	P0012S
Trizol	Thermo Fisher	15596026CN
HiScript III RT SuperMix for qPCR	Vazyme	R323-01
AceQ Universal SYBR qPCR Master Mix	Vazyme	Q511-02

Supplementary Table S2: List of primary and secondary antibodies.

Antibodies	Manufacturer	Catalog number	Dilution	Host
Primary antibodies				
RPE65	Abclonal	A9615	1:1000 (WB)	Rabbit
TJP1	Proteintech	66452-1-Ig	1:200 (IF)	Mouse
RHO	Bioss	bs-19872R	1:200 (IF)	Rabbit
BAX	abcam	ab32503	1:1000 (WB)	Rabbit
NOX2	Proteintech	19013-1-AP	1:200 (IF)	Rabbit
IBA1	HUABIO	ET1705-78	1:1000 (WB)	Rabbit
IBA1	HUABIO	HA601368	1:200 (IF)	Guinea pig
CD68	HUABIO	HA722285	1:200 (IF)	Rabbit
GFAP	HUABIO	EM140707	1:1000 (WB)	Mouse
GFAP	Proteintech	16825-1-AP	1:200 (IF)	Rabbit
CASP3	CST	9664	1:200 (IF)	Rabbit
Secondary antibodies				
Goat anti-Rabbit IgG 488	Thermo Fisher	A32731	1:500 (IF)	
Goat anti-Rabbit IgG594	Bioss	bs-0295G-AF594	1:500 (IF)	
Goat anti-mouse	Bioss	bs-0296G-	1:500 (IF)	

IgG 488	AF488

Supplementary Table S3: sequences of primers.

Gene	Forward	Reverse
Gapdh	AGGTCGGTGTGAACGGATTTG	TGTAGACCATGTAGTTGAGGTCA
Gpx1	GTTTCCCGTGCAATCAGTTC	CAATGTAAAATTGGGCTCGAA
Trp53	CCCCTGTCATCTTTTGTCCCT	AGCTGGCAGAATAGCTTATTGAG
Casp3	CTGACTGGAAAGCCGAAACTC	CGACCCGTCCTTTGAATTTCT
Casp7	GGACCGAGTGCCCACTTATC	TCGCTTTGTCGAAGTTCTTGTT
Casp8	CAACTTCCTAGACTGCAACCG	TCCAACTCGCTCACTTCTTCT
Mlkl	ACTGTGAACTTGGAACCCTG	TGCTGATGTTTCTGTGGAGTG
Tradd	ACGAACTCACTAGTCTAGCAGAG	AATACCCCAACAGCCACC
Ripk3	AAGGAAGCCACACCAAGATC	AGTTCCCAATCTGCACTTCAG
Ripk1	GGAAGGATAATCGTGGAGGC	AAGGAAGCCACACCAAGATC
Tnfrsf1a	CTCTGCTCTACGAATCACTCTG	CACAGCATACAGAATCGCAAG

Il1b	TGGACCTTCCAGGATGAGGACA	GTTCATCTCGGAGCCTGTAGTG
Tnf	AGACCCTCACACTCAGATCATCTTC	TTGCTACGACGTGGGCTACA
Csf1	GCCTCCTGTTCTACAAGTGGAAG	ACTGGCAGTTCCACCTGTCTGT
Ccl2	CAGGTCCCTGTCATGCTTCT	GTGGGGCGTTAACTGCATCT
Il6	TAGTCCTTCCTACCCCAATTTCC	TTGGTCCTTAGCCACTCCTTC
Il4	GGTCTCAACCCCCAGCTAGT	GCCGATGATCTCTCTCAAGTGAT
Il10	CGGGAAGACAATAACTGCACCC	CGGTTAGCAGTATGTTGTCCAGC
Il13	CCTGGCTCTTGCTTGCCTT	GGTCTTGTGTGATGTTGCTCA
Tgfb1	TGATACGCCTGAGTGGCTGTCT	CACAAGAGCAGTGAGCGCTGAA
Fgf2	AAGCGGCTCTACTGCAAGAACG	CCTTGATAGACACAACTCCTCTC
Ctsb	AGACCTGCTTACTTGCTGTG	GGAGGGATGGTGTATGGTAAG
Gpx1	GTTTCCCGTGCAATCAGTTC	CAATGTAAAATTGGGCTCGAA
Gss	GATCCTGTCCAATAACCCCAG	GCACGCTGGTCAAATATGTTC
Hmox1	GAGCCTGAATCGAGCAGAAC	CCTTCAAGGCCTCAGACAAA
Nrf2	GATGAGGATGGAAAGCCTTAC	TAGCTCAGAAAAGGCTCCATC

Ncf1	TCATCCTTCAGACCTATCGGG	ACCTCGCTTTGTCTTCATCTG
Ncf2	GCAGAAGAGCAGTTGGCATTG<br>G	CTGCCTCTCATTTGGACGGAAC
Nox2	TGGCGATCTCAGCAAAAGGTGG	GTACTGTCCCACCTCCATCTTG
Nox4	TCCAAGCTCATTTCCCACAG	CGGAGTTCCATTACATCAGAGG
Gstp1	GGATATGGTGAATGATGGGG	GGGCCTTCACGTAGTCATTC
