# Supplementary material for: Intermittent fasting attenuates glial hyperactivation and photoreceptor degeneration in a NaIO3-induced mouse model of age-related macular degeneration
Source: Commun Biol. 2025 Oct 1;8:1408. doi: 10.1038/s42003-025-08815-0 (PMC12488855; doi:10.1038/s42003-025-08815-0)
Supplement: Supplementary file 3 — Description of Additional Supplementary Files [file 42003_2025_8815_MOESM3_ESM.pdf]

Description of Additional Supplementary Files

File name: Supplementary Data 1

Description: The source data behind the graphs.

File name: Supplementary Data 2

Description: The source data of qPCR.
