# Supplementary material for: Intermittent fasting attenuates glial hyperactivation and photoreceptor degeneration in a NaIO3-induced mouse model of age-related macular degeneration
Source: Commun Biol. 2025 Oct 1;8:1408. doi: 10.1038/s42003-025-08815-0 (PMC12488855; doi:10.1038/s42003-025-08815-0)
Supplement: Supplementary file 6 — Reporting Summary [file 42003_2025_8815_MOESM6_ESM.pdf]

nature portfolio

Corresponding author(s): Meng Li

Last updated by author(s): Jul 18, 2025

Reporting Summary

Nature Portfolio wishes to improve the reproducibility of the work that we publish. This form provides structure for consistency and transparency in reporting. For further information on Nature Portfolio policies, see our Editorial Policies and the Editorial Policy Checklist.

Statistics

For all statistical analyses, confirm that the following items are present in the figure legend, table legend, main text, or Methods section.

n/a	Confirmed	
☐	☒	The exact sample size (*n*) for each experimental group/condition, given as a discrete number and unit of measurement
☐	☒	A statement on whether measurements were taken from distinct samples or whether the same sample was measured repeatedly
☐	☒	The statistical test(s) used AND whether they are one- or two-sided<br>*Only common tests should be described solely by name; describe more complex techniques in the Methods section.*
☒	☐	A description of all covariates tested
☐	☒	A description of any assumptions or corrections, such as tests of normality and adjustment for multiple comparisons
☐	☒	A full description of the statistical parameters including central tendency (e.g. means) or other basic estimates (e.g. regression coefficient) AND variation (e.g. standard deviation) or associated estimates of uncertainty (e.g. confidence intervals)
☐	☒	For null hypothesis testing, the test statistic (e.g. *F*, *t*, *r*) with confidence intervals, effect sizes, degrees of freedom and *P* value noted<br>*Give P values as exact values whenever suitable.*
☒	☐	For Bayesian analysis, information on the choice of priors and Markov chain Monte Carlo settings
☒	☐	For hierarchical and complex designs, identification of the appropriate level for tests and full reporting of outcomes
☐	☒	Estimates of effect sizes (e.g. Cohen's *d*, Pearson's *r*), indicating how they were calculated

Our web collection on statistics for biologists contains articles on many of the points above.

Software and code

Policy information about availability of computer code

Data collection	All codes used in this study can be provided by the corresponding author upon request
Data analysis	All codes used in this study can be provided by the corresponding author upon request

For manuscripts utilizing custom algorithms or software that are central to the research but not yet described in published literature, software must be made available to editors and reviewers. We strongly encourage code deposition in a community repository (e.g. GitHub). See the Nature Portfolio guidelines for submitting code & software for further information.

Data

Policy information about availability of data

All manuscripts must include a data availability statement. This statement should provide the following information, where applicable:

- Accession codes, unique identifiers, or web links for publicly available datasets
- A description of any restrictions on data availability
- For clinical datasets or third party data, please ensure that the statement adheres to our policy

All data needed to replicate our results are available in the article, on-line or the supplementary information

Research involving human participants, their data, or biological material

Policy information about studies with human participants or human data. See also policy information about sex, gender (identity/presentation), and sexual orientation and race, ethnicity and racism.

Reporting on sex and gender	Does not apply
Reporting on race, ethnicity, or other socially relevant groupings	Does not apply
Population characteristics	Does not apply
Recruitment	Does not apply
Ethics oversight	Does not apply

Note that full information on the approval of the study protocol must also be provided in the manuscript.

Field-specific reporting

Please select the one below that is the best fit for your research. If you are not sure, read the appropriate sections before making your selection.

☒ Life sciences ☐ Behavioural & social sciences ☐ Ecological, evolutionary & environmental sciences

For a reference copy of the document with all sections, see nature.com/documents/nr-reporting-summary-flat.pdf

Life sciences study design

All studies must disclose on these points even when the disclosure is negative.

Sample size	The specific sample sizes are detailed in each figure legend. Briefly, a sample size of n=3 was used for WB, qPCR and RNA-seq, while at least 5 samples per group were used for immunostaining and other related experiments. This was deemed sufficient due to the use of internal controls (specific staining of positionally defined cell types using known markers) and the low observed variability between stained samples.
Data exclusions	Data were not excluded from analysis.
Replication	All replication attempts were successful, and the observed marker expression pattern was consistent with previously known results from orthogonal approaches. For the final quantification, at least three discontinuous retinal sections were analyzed for each mouse, while four regions from the same central area were examined for the whole mount samples from each mouse.
Randomization	A computer-generated random sequence was utilized to allocate all experimental animals into various treatment groups. This randomization occurred prior to the commencement of the experiment to ensure group balance and minimize potential biases.
Blinding	The OMR was analyzed and recorded in a blinded manner. After collecting the OMR videos an independent individual, unaware of the animal groupings, conducted the statistical analysis of the animals' visual behavior. Blinded statistics were not applicable to other experiments.

Reporting for specific materials, systems and methods

We require information from authors about some types of materials, experimental systems and methods used in many studies. Here, indicate whether each material, system or method listed is relevant to your study. If you are not sure if a list item applies to your research, read the appropriate section before selecting a response.

Materials & experimental systems

n/a	Involved in the study	
☐	☒	Antibodies
☒	☐	Eukaryotic cell lines
☒	☐	Palaeontology and archaeology
☐	☒	Animals and other organisms
☒	☐	Clinical data
☒	☐	Dual use research of concern
☒	☐	Plants

Methods

n/a	Involved in the study	
☒	☐	ChIP-seq
☒	☐	Flow cytometry
☒	☐	MRI-based neuroimaging

Antibodies

Antibodies used	The full list is also available in supplementary information, Supplementary Table S3.<br>Primary antibodies:<br>RPE65: Abclona, A9615, ARC1659 (clone), 4000001659 (Lot)<br>ZO-1: Proteintech, 66452-1-Ig, 1G4A1 (clone), 10019107 (Lot)<br>Rhodopsin : Bioss, bs-19872R, N (clone), BJ01074684 (Lot)<br>BAX: abcam, ab32503, E63 (clone), GR3275117 (Lot)<br>NOX2: Proteintech, 19013-1-AP, N(clone), 00045148 (Lot)<br>IBA1: HUABIO, ET1705-78, JM36-62 (clone), H651006031 (Lot)<br>GFAP: HUABIO, EM140707, 1-D4 (clone), HO0604 (Lot)<br>GFAP: Proteintech, 16825-1-AP, N(clone), 00163293 (Lot)<br>Cleaved Caspase-3: CST, 9664, 5A1E (clone), 22 (Lot)<br>CD68: HUABIO, HA722285, PSH05-47 (clone), H680619010<br>Secondary antibodies:<br>Goat anti-Rabbit IgG 488, Thermo Fisher, A11008, N (clone), 2743033 (Lot)<br>Goat anti-Rabbit IgG594, Bioss, bs-0295G-AF594, N (clone), BA12206761 (Lot)<br>Goat anti-mouse IgG 488, Bioss, bs-0296G-AF488, N (clone), BA12206277 (Lot)
Validation	All antibodies used are sourced from commercial suppliers as described. Only antibodies validated by the vendor through in vitro and in situ experiments (for IHC, IF, and WB, with images available on their websites) and/or widely used by the scientific community with multiple references were selected. The validation details and references for each antibody are publicly available on the respective vendor websites, accessible via the catalog numbers listed above.

Animals and other research organisms

Policy information about studies involving animals; ARRIVE guidelines recommended for reporting animal research, and Sex and Gender in Research

Laboratory animals	Eight-week-old male C57BL/6J mice (body weight: 22–24 g) were sourced from Cavens Laboratory Animal Co., Ltd. (Changzhou, China). Middle-aged (9-month-old) and aged (13-month-old) male C57BL/6J mice were obtained from the Animal Center of Xuzhou Medical University. All mice were caged under specific pathogen-free (SPF) conditions in temperature-controlled facilities maintained at 22–25 °C with a 12-hour light/dark cycle.
Wild animals	The study did not involve wild animals.
Reporting on sex	The disease studied is macular degeneration. Previous studies have not reported any gender differences in AMD among humans. Therefore, based on these studies, male mice were predominantly chosen, and only male mice were included in this study.
Field-collected samples	The study did not involve samples collected from the field.
Ethics oversight	All animal-related experimental procedures were conducted in accordance with the ARVO Statement for the Use of Animals in Ophthalmic and Vision Research and were approved by the Animal Ethics Committee of Xuzhou Medical University. (Ethic ID: 202306T029).

Note that full information on the approval of the study protocol must also be provided in the manuscript.

Plants

Seed stocks	*Report on the source of all seed stocks or other plant material used. If applicable, state the seed stock centre and catalogue number. If plant specimens were collected from the field, describe the collection location, date and sampling procedures.*
Novel plant genotypes	*Describe the methods by which all novel plant genotypes were produced. This includes those generated by transgenic approaches, gene editing, chemical/radiation-based mutagenesis and hybridization. For transgenic lines, describe the transformation method, the number of independent lines analyzed and the generation upon which experiments were performed. For gene-edited lines, describe the editor used, the endogenous sequence targeted for editing, the targeting guide RNA sequence (if applicable) and how the editor was applied.*
Authentication	*Describe any authentication procedures for each seed stock used or novel genotype generated. Describe any experiments used to assess the effect of a mutation and, where applicable, how potential secondary effects (e.g. second site T-DNA insertions, mosiacism, off-target gene editing) were examined.*
